# The impact of anti-phosphatidylserine/prothrombin antibodies on pregnancy outcomes in patients with unexplained recurrent implantation failure: a retrospective cohort study

**DOI:** 10.3389/fimmu.2026.1731905

**Published:** 2026-01-21

**Authors:** Donglin Han, Jiansuo Zhou, Jing Shi, Nan Yang, Qiong Liu, Jun Zhang, Haiyan Wang, Yang Wang, Jie Zhao

**Affiliations:** 1 Center for Reproductive Medicine, Department of Obstetrics and Gynecology, Peking University Third Hospital, Beijing, China; 2 Key Laboratory of Assisted Reproduction (Peking University), Ministry of Education, Beijing, China; 3 Department of Laboratory Medicine, Peking University Third Hospital, Beijing, China; 4 Department of Pharmacy, Peking University Third Hospital, Beijing, China; 5 Blood Transfusion Department, Peking University Third Hospital, Beijing, China; 6 National Clinical Research Center for Obstetrics and Gynecology (Peking University Third Hospital), Beijing, China; 7 State Key Laboratory of Female Fertility Promotion, Department of Obstetrics and Gynecology, Peking University Third Hospital, Beijing, China

**Keywords:** anti-phosphatidylserine prothrombin antibodies, *in vitro* fertilization (IVF)/intracytoplasmic sperm injection (ICSI), live birth, pregnancy outcomes, unexplained recurrent implantation failure (URIF)

## Abstract

**Background:**

Unexplained recurrent implantation failure (URIF) represents a complex and challenging condition in reproductive medicine, while the role of non-criteria antiphospholipid syndrome antibodies in its pathogenesis remains unclear. As a non-criteria antiphospholipid syndrome antibody, anti-phosphatidylserine/prothrombin antibodies were investigated in this study to explore their effects on oocyte quality and pregnancy outcomes in patients with unexplained recurrent implantation failure.

**Methods:**

This was a retrospective cohort study that included 853 IVF/ICSI cycles from patients with URIF at Peking University Third Hospital between April 2021 and April 2023, with patients stratified into positive and negative groups for anti-phosphatidylserine/prothrombin antibodies. Propensity score matching (PSM) was conducted at a 1:3 ratio to adjust for potential confounding factors. The primary outcome was the cumulative live birth rate, and secondary outcomes included the live birth rate per transfer cycle, miscarriage rate, and oocyte quality.

**Results:**

After PSM, no significant differences were detected between the aPS/PT-positive and negative groups in terms of the oocyte and embryo parameters. Compared with the aPS/PT-negative group, the positive group presented a lower cumulative live birth rate (20% vs 32.1%, p<0.05) and a higher miscarriage rate (41.4% vs 20.4%, p<0.05). After adjusting for confounding factors via binary logistic regression (for miscarriage) and multivariate Cox models (for cumulative live birth), the results revealed that aPS/PT antibody positivity may be a potential independent risk factor for miscarriage in patients with URIF (OR = 3.753, 95% CI: 1.297-10.854, p<0.05), but its correlation with cumulative live birth rate was not significant (HR = 0.391, 95% CI: 0.391-1.179, p=0.170).

**Conclusion:**

Anti-phosphatidylserine/prothrombin antibody (aPS/PT) positivity may be a risk factor for increased miscarriage rates in patients with unexplained recurrent implantation failure.

## 1 Introduction

In the reproductive field, recurrent implantation failure (RIF) continues to be a crucial area of study, encompassing multifactorial etiologies and diverse therapeutic approaches. The current diagnostic criteria for RIF lack universal standardization. Notably, the European Society of Human Reproduction and Embryology (ESHRE) Preimplantation Genetic Diagnosis Consortium has established a consensus definition: failure to achieve clinical pregnancy following either 1) the transfer of ≥3 morphologically high-grade embryos in fresh or frozen-thawed cycles, or 2) the cumulative transfer of ≥10 embryos across multiple ovarian stimulation cycles (1). According to these criteria, epidemiological data from two university medical centers in the Netherlands demonstrated an 8% incidence rate of RIF among women aged <39 years (2). Coughlan proposed a more widely accepted definition of RIF, specifying the condition as failure to attain clinical pregnancy in women <40 years of age following ≥3 sequential cycles of fresh or frozen embryo transfer cycles with cumulative transfer of ≥4 morphologically optimal embryos (3). Despite extensive investigations into the etiology of RIF, approximately 50% of patients remain classified as unexplained recurrent implantation failure (URIF) (4). Embryo implantation in assisted reproductive technology (ART) is a complex process influenced by multiple factors, including age, body mass index (BMI), embryo quality, the uterine environment, endocrine and metabolic diseases, immune disorders, autoimmunity, genetic factors, and lifestyle (4). Notably, the pathophysiological role of autoantibodies in implantation failure has emerged as a pivotal research frontier. Scientific investigations have revealed that antiphospholipid antibodies (aPLs) can directly bind to trophoblast cells via β2-glycoprotein I (β2GPI) expressed on their surface, thereby triggering localized inflammatory responses, impairing trophoblast proliferation, invasion, and syncytialization, and inhibiting the secretion of human chorionic gonadotropin (hCG) from placental tissue (5, 6). On the other hand, emerging data suggest that aPLs may drive endometrial microthrombosis, thereby inducing impaired decidualization and subsequent compromise of endometrial receptivity, potentially culminating in embryo implantation failure (7, 8).

Anti-phosphatidylserine/prothrombin antibodies (aPS/PT), a distinct subgroup within aPLs, were initially characterized in 2000 by Atsumi Tatsuya and associates. These autoantibodies specifically recognize the phosphatidylserine-prothrombin complex (9), demonstrating prothrombotic properties through the stimulation of both endothelial cells and platelets (10). Compared with other non-standard aPLs, aPS/PT has superior clinical relevance, among patients with systemic lupus erythematosus (SLE), the concentration of aPS/PT antibodies demonstrated the strongest association with arterial thrombosis (aOR = 3.47, 95% CI: 2.13–5.63) and was independently associated with recurrent pregnancy loss (aOR = 2.38, 95% CI: 1.42–4.00) (11). And aPS/PT can synergistically enhance the production of tissue factor (TF) and proinflammatory cytokine (TNF-α) in monocytes. TF subsequently activates the coagulation cascade (12), whereas TNF-α impairs uterine spiral arteries and inhibits placental differentiation and invasion (13), ultimately leading to implantation failure. Notably, aPS/PT is related to various autoimmune clinical symptoms and may be a risk factor for thrombosis (14–16), early miscarriage, and fetal demise (beyond 10 weeks of gestation) (17). A multivariate analysis of 186 antiphospholipid syndrome (APS) cohorts demonstrated significant associations between aPS/PT and thrombosis (OR = 6.72) as well as adverse gestational outcomes (particularly miscarriage) (OR = 9.44), when standard APS antibody tests yield negative results, aPS/PT may serve as a potential risk predictor for venous thrombosis and obstetric complications (16). However, the effect of aPS/PT on early embryonic maintenance remains undetermined, with limited clinical evidence available, specifically regarding its effects on reproductive outcomes in patients who have unexplained recurrent implantation failure (URIF). Our study aimed to assess the effects of aPS/PT antibodies on oocyte quality and pregnancy outcomes in URIF patients.

## 2 Materials and methods

### 2.1 Study cohorts and classification

**Figure 1:**
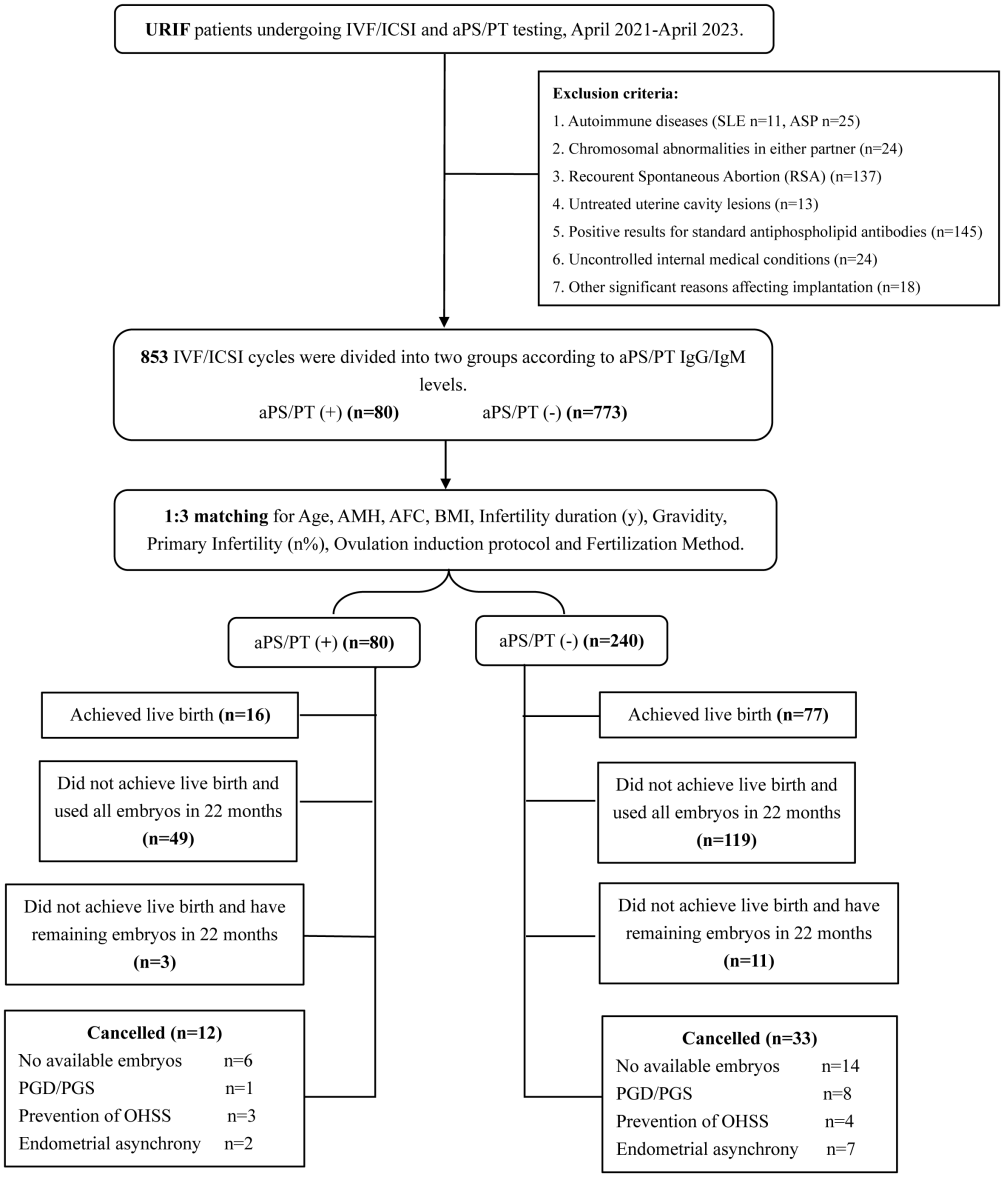
Study flowchart. URIF, Unexplained Recurrent Implantation Failure; IVF, In Vitro Fertilization; ICSI, Intracytoplasmic Sperm Injection; aPS/PT, Anti-Phosphatidylserine/Prothrombin antibodies; AMH, Anti-Müllerian Hormone; BMI, Body Mass Index; AFC, Antral Follicle Count; PGD/PGS, Preimplantation Genetic Diagnosis/Preimplantation Genetic Screening; OHSS, Ovarian Hyperstimulation Syndrome.

This study was approved by the Ethics Committee of Peking University Third Hospital (Approval No: M20241083), and the clinical data of URIF patients who underwent IVF/ICSI treatment at Peking University Third Hospital between April 2021 and April 2023 were retrospectively analyzed. The dataset included patients’ baseline characteristics, pregnancy outcomes, and prior examination records, including: 1) couple karyotyping; 2) color Doppler ultrasonography; 3) autoimmune profiling (such as Systemic Lpus Erythematosus [SLE], Thrombophilia, Systemic Sclerosis [SSc], Rheumatoid Arthritis [RA], and Anti-phospholipid Syndrome [APS]). 4) Follow-up records of pregnancy outcomes within 2 years after oocyte retrieval. The study included 853 URIF cases, with 80 aPS/PT positive and 773 negative cases. All participants had complete medical histories and received standardized medical follow-up. The flowchart is detailed in (**Figure 1**).

Anti-phosphatidylserine/prothrombin antibodies (aPS/PT) are included as one of the routine immunological screening indicators at our center and have been incorporated into the pre-treatment evaluation for infertility patients undergoing *in vitro* fertilization-embryo transfer (IVF-ET/FET). The routine detection of aPS/PT IgG/IgM at our institution was performed using commercial enzyme-linked immunosorbent assay (ELISA) kits (QUANTA Lite aPS/PT IgG ELISA, INOVA Diagnostics, Inc., San Diego, CA, USA, Cat. No. 708835; and QUANTA Lite aPS/PT IgM ELISA, INOVA Diagnostics, Inc., San Diego, CA, USA, Cat. No. 708845). Patients undergo this assay within two months prior to initiating IVF/ICSI treatment. The positivity threshold was defined as 30 IU/mL. A single detection of either aPS/PT IgG or IgM exceeding 30 IU/mL is classified as initial screening positivity. The grouping criteria for this study was the positive group: which was defined by two consecutive positive test results spaced 12 weeks apart. The negative group: which was defined by results ≤30 IU/mL at both time points.

The inclusion criteria were as follows: 1) Aged 20–40 years. 2) Diagnosis of recurrent implantation failure (RIF) (18), described as implantation failure after two sequential cycles of frozen embryo replacement, IVF, or ICSI, cumulatively with: ≥4 transferred cleavage-stage embryos or ≥2 blastocysts, embryos meeting morphologically good-quality criteria and developmentally appropriate staging. 3) Patients who underwent autoimmune disease screening and aPS/PT testing during clinical evaluation.

The exclusion criteria were as follows: 1) Autoimmune disorders, such as rheumatoid arthritis (RA), connective tissue disease, dry eye syndrome (keratoconjunctivitis sicca), systemic lupus erythematosus (SLE), thrombophilia and antiphospholipid syndrome (APS). 2) Chromosomal abnormalities in either partner. 3) Diagnosis of Recurrent Spontaneous Abortion (RSA). 4) Untreated uterine cavity abnormalities, such as: thin endometrium (thickness < 6 mm), uterine malformations (unicornuate uterus, bicornuate uterus), intrauterine adhesions, submucosal fibroids and large adenomyosis (diameter > 8 cm). 5) Positive for standard antiphospholipid antibodies (aCL/anti-β2GPI). 6) Positive lupus anticoagulant (LAC) and related antiphospholipid antibody coagulation assays, including the dilute Russell’s viper venom time (dRVVT), silica clotting time (SCT), and anti-phospholipid antibody test panel (APLTA). 7) Uncontrolled systemic diseases, including endocrine disorders (thyroid dysfunction, diabetes mellitus, etc.).

### 2.2 Observation indicators and data acquisition

Clinical data were extracted from medical records, including the following: 1) Demographics: age, BMI, infertility period, gravidity and parity. 2) Biomarkers of ovarian response: antral follicle count (AFC), baseline hormone levels (e.g., bFSH, bLH, bE2), anti-Müllerian (AMH). 3) Immunological indicators: Data on the expression of patients’ autoantibodies, including standard antiphospholipid antibodies (e.g., anti-cardiolipin antibodies [aCLs], anti-β2-glycoprotein I antibodies [β2-GPI], and lupus anticoagulant [LAs]), antinuclear antibodies (ANAs), anti-thyroid antibodies (anti-thyroid peroxidase antibodies [TPOAbs] and anti-thyroglobulin antibodies [TgAbs]), and aPS/PT. 4) Ovulation stimulation outcomes: no. of oocytes retrieved, no. of 2 pronuclei (2PN) embryos, no. of high-quality embryos, no. of embryos transferred. 5) Pregnancy outcome: Embryo transfer data from enrolled oocyte retrieval cycles performed within two years after ovarian stimulation were extracted from the hospital follow-up system. All data in this study were sourced from the electronic medical record (EMR) system and cycle follow-up tracking system of Peking University Third Hospital.

The primary outcome was the cumulative live birth rate (CLBR), which requires achieving live birth within 2 years after ovarian stimulation. The secondary outcomes included the number of oocytes retrieved, number of transferable embryos, fertilization rate, 2PN rate, live birth rate per transfer cycle, embryo implantation rate, biochemical pregnancy rate, clinical pregnancy rate, miscarriage rate, and cancellation rate. Live birth was defined as the delivery of a viable infant after 22 weeks of gestation. Cumulative live birth rate = (no. of live births/no. of oocyte retrieval cycles) × 100%; Live birth rate per transfer cycle = (no. of live births/no. of transfer cycles) × 100%; Embryo implantation rate = (no. of gestational sacs observed under ultrasound at 4–5 weeks/total no. of embryos transferred) × 100%; Biochemical pregnancy rate = (no. of cycles with biochemical pregnancy/total no. of embryo transfer cycles) × 100%; Clinical pregnancy was defined as the detection of at least one gestational sac by ultrasound at 6 weeks of gestation, with clinical pregnancy rate = (no. of clinical pregnancies/no. of transfer cycles) × 100%; Miscarriage rate = (no. of miscarriages before 22 weeks/no. of clinical pregnancies) × 100%; Cancellation rate = (no. of canceled cycles/no. of oocyte retrieval cycles) × 100%; Fertilization rate = (no. of fertilized oocytes/total no. of oocytes retrieved) × 100%; 2PN rate = (no. of 2PN embryos/no. of MII oocytes) × 100%; Cleavage rate = (no. of cleaved embryos/total no. of fertilized oocytes) × 100%; High-quality embryos rate = (no. of high-quality embryos/total no. of embryos) × 100%.

### 2.3 Statistical methods

To minimize potential confounding effects, this study employed propensity score matching (PSM) for covariate adjustment via nearest neighbor matching at a 1:3 ratio without replacement with a caliper value of 0.05, with covariates including age, BMI, AMH, infertility duration, infertility type, antral follicle count, ovulation induction protocol, and fertilization method. Continuous variables that were normally distributed were compared using Student’s t-test and were expressed as the means ± standard deviations (x̄ ± s), while non-normally distributed variables were compared using the Mann-Whitney U test and were expressed as medians with interquartile ranges. Categorical variables were analyzed using the chi-square test (χ^2^ test) and were presented as frequencies (percentages). Kaplan-Meier curves were used to visually compare the cumulative pregnancy rate over time between the antibody-positive and negative groups, with differences assessed by the log-rank test. A binary logistic regression model was employed to evaluate the impact of potential confounding factors on the live birth rate per transfer and miscarriage rate, and a Cox proportional hazards model was used to assess the relative prognostic significance of aPS/PT antibodies and potential confounding factors on the cumulative live birth rate. Statistical analyses were performed using SPSS 24.0 and GraphPad Prism 10 software.

## 3 Results

Ultimately, a total of 791 patients with unexplained recurrent implantation failure (URIF) were included in the analysis, comprising 73 aPS/PT-positive patients and 718 aPS/PT-negative patients. This study selected oocyte retrieval cycles as the unit of analysis, encompassing a total of 853 cycles. Among these, 80 cycles were in the aPS/PT-positive group and 773 cycles in the aPS/PT-negative group. To adjust for confounding factors, propensity score matching at a 1:3 ratio was performed, resulting in the inclusion of 240 cycles from the negative group (derived from 230 aPS/PT-negative patients) for subsequent analysis. The number of cycles with remaining embryos that did not achieve live birth within 2 years was 3 in the positive group and 11 in the negative group (**Figure 1**).

### 3.1 Baseline characteristics

Prior to PSM, significant differences were observed between the aPS/PT-positive and negative groups in terms of primary infertility (n%), AMH, antral follicle count, and fertilization method (P<0.05). To ensure data comparability, PSM was performed at a 1:3 ratio without replacement for these variables along with age, BMI, infertility duration, and ovulation induction protocol. Following PSM, the baseline characteristics were balanced between the positive and negative groups (P > 0.05, with no statistically significant differences observed), although 553 oocyte retrieval cycles were excluded, resulting in a significant reduction in the number of cycles in the antibody-negative group, this process selectively retained well-matched cycles to achieve covariate balance between groups, effectively controlling for potential confounding factors and establishing a well-comparable cohort for subsequent analysis. Ultimately, the analysis included 80 oocyte retrieval cycles in the positive group and 240 matched oocyte retrieval cycles in the negative group. (**Table 1**).

**Table 1:** Baseline characteristics of oocyte retrieval cycles in patients with URIF: aPS/PT-positive vs. negative groups.

Characteristic	Antibody positive group (n=80)	Antibody negative group (n=773)	P1-value	Antibody negative group after PSM matching (n=240)	P2-value
Age (y)	35.53 ± 4.25	35.22 ± 4.50	0.565	35.40 ± 4.29	0.821
BMI (kg/m^2^)	21.9 (20.0-24.4)	20.8 (20.0-24.3)	0.865	21.5 (20.0-24.3)	0.651
Infertility duration (y)	4.5 (3.0-7.0)	4.0 (3.0-7.0)	0.839	4.0 (3.0-7.0)	0.727
Primary infertility (n%)	46 (57.5%)	546 (70.6%)	<0.05	147 (61.3%)	0.5553
Gravidity	0.66 ± 1.10	0.42 ± 0.795	0.054	0.58 ± 0.88	0.471
AMH (ng/ml)	2.07 ± 1.77	2.93 ± 2.90	<0.05	2.15 ± 1.66	0.708
bFSH (mIU/ml)	5.15 (3.39-6.61)	5.79 (4.00-7.55)	0.054	5.97 (3.14-7.59)	0.122
bLH (mIU/ml)	3.20 ± 9.36	4.22 ± 14.20	0.533	3.79 ± 12.62	0.704
bE2 (mIU/ml)	123.5 (58.5-199.8)	130.5 (89.3-178.0)	0.455	126.0 (76.0-171.0)	0.833
AFC	8 (4-11)	9 (6-13)	<0.05	8 (6-11)	0.189
Indications for IVF					
Pelvic and tubal factors (n%)	31 (38.8%)	252 (32.6%)		92 (38.3%)	
Endometriosis (n%)	2 (2.5%)	44 (5.7%)		16 (6.7%)	
Ovulation disorders (n%)	13 (16.3%)	95 (12.3%)	0.386	22 (9.2%)	0.334
Male factor (n%)	6 (7.5%)	85 (11.0%)		18 (7.5%)	
Unexplained (n%)	28 (35.0%)	297 (38.4%)		92 (38.3%)	
Fertilization method					
IVF(n%)	49 (61.3%)	379 (49.0%)	<0.05	143 (59.6%)	0.792
ICSI (n%)	31 (38.8%)	394 (51.0%)		97 (40.4%)	

Continuous variables were presented as mean ± standard deviation and median-interquartile range, while categorical variables were expressed as number (percentage), p< 0.05 was considered statistically significant. P1: antibody positive group(n=80) vs antibody negative group (n=773). P2: antibody positive group (n=80) vs antibody negative group after PSM matching (n=240). Post-PSM, potential confounders showed comparable distributions (P2 > 0.05), indicating successful control of confounding bias.

BMI, Body Mass Index; AMH, Anti-Müllerian Hormone; bFSH, basal Follicle-Stimulating Hormone; bLH, basal Luteinizing Hormone; bE2, basal Estradiol; AFC, Antral Follicle Count; IVF, In Vitro Fertilization; ICSI, Intracytoplasmic Sperm Injection.

### 3.2 Ovulation stimulation outcomes and embryological characteristics

Compared with the negative group, the cleavage rate in the antibody-positive group was lower (96.9% vs 98.2%, p=0.063), although the difference was not statistically significant. Additionally, no significant differences were observed in the number of oocytes yield, fertilization count/rate, 2PN count/rate, number/rate of high-quality embryos, or number of transferable embryos between the two groups (**Supplementary Table S1**).

### 3.3 Pregnancy outcomes per transfer cycle and cumulative live birth rate

**Figure 2:**
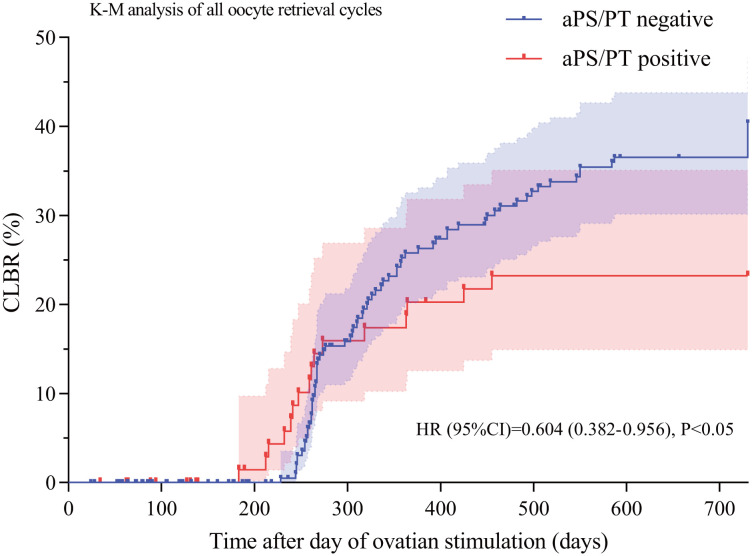
Kaplan-Meier curves of cumulative live birth rate (CLBR) per oocyte retrieval cycle in aPS/PT-positive and aPS/PT-negative groups. Kaplan-Meier curve was used to visually compare the cumulative pregnancy rate per oocyte retrieval cycle over time between the antibody-positive and negative groups, with intergroup differences assessed by the log-rank test, p<0.05 was considered statistically significant. Abbreviations: CI, confidence interval; KM, Kaplan-Meier method; HR, hazard ratio; aPS/PT, Anti-Phosphatidylserine/Prothrombin antibodies.

During the 2-year follow up period, the antibody-positive group underwent 75 transfer cycles (including 36 fresh cycles and 39 frozen-thawed embryo transfer cycles), whereas the antibody-negative group underwent 257 transfer cycles (including 116 fresh cycles and 141 frozen-thawed embryo transfer cycles). Univariate analysis revealed that, compared with the antibody-negative group, the positive group showed a significantly lower cumulative live birth rate per oocyte retrieval cycle (20.0% vs 32.1%, p<0.05) and a significantly higher miscarriage rate per transfer cycle (41.4% vs 20.4%, p<0.05) (**Table 2**). Kaplan-Meier analysis also demonstrated that the cumulative incidence of achieving an ongoing pregnancy culminating in live birth per oocyte retrieval cycle was significantly lower in the antibody-positive group compared to the antibody-negative group (HR (95% CI) = 0.604 (0.382–0.956), p < 0.05) (**Figure 2**).

**Table 2:** Pregnancy and cumulative between the 2 groups after propensity score matching.

Characteristic	Antibody positive group	Antibody negative group after PSM matching	P-value	Risk ratio (95% CI)
No. of oocyte retrieval cycles (n)	80	240	—	—
Total no. of transfer cycles (n)	75	257	—	—
Cycle type of embryo transferred, n (%)				
ET	36 (48.0)	116 (45.1)	0.661	1.063 (0.811-1.395)
FET	39 (52.0)	141 (54.9)		0.948 (0.743-1.210)
No. of embryos transferred, n (%)				
1	34 (45.3)	103 (40.1)	0.416	1.131 (0.846-1.512)
2	41 (54.7)	154 (59.9)		0.912 (0.726-1.147)
Embryo transfer stage, n (%)				
Cleavage stage	52 (69.3)	184 (71.6)	0.704	0.968 (0.818-1.147)
Blastocyst	23 (30.7)	73 (28.4)		1.080 (0.730-1.597)
Endometrial preparation, n (%)				
Natural cycles	15 (38.5)	41 (29.1)	0.261	1.323 (0.824-2.123)
Ovarian stimulation cycles	7 (17.9)	18 (12.8)		1.406 (0.633-3.122)
HRT Cycles	17 (43.6)	82 (58.6)		0.750 (0.511-1.100)
Pregnancy outcome, n (%)				
Cumulative live birth rate	16 (20.0)	77 (32.1)	<0.05	0.623 (0.388-1.003)
Live birth rate per transfer	16 (21.3)	77 (29.9)	0.143	0.712 (0.444-1.143)
Embryo implantation rate	34/150 (22.7)	110/521 (21.1)	0.683	1.074 (0.765-1.507)
Biochemical pregnancy rate	33 (44.0)	112 (43.6)	0.949	1.010 (0.755-1.350)
Clinical pregnancy rate	29 (38.7)	95 (37.0)	0.789	1.046 (0.755-1.450)
Miscarriage rate	12 (41.4)	19 (20.4)	<0.05	2.069 (1.146-3.737)

Categorical variables were analyzed using the chi-square test (χ^2^ test) and expressed as frequencies (percentages), p< 0.05 was considered statistically significant.

Cumulative live birth rate = (no. of live births/no. of oocyte retrieval cycles) × 100%; Live birth rate per transfer cycle = (no. of live births/no. of transfer cycles) × 100%; Embryo implantation rate = (no. of gestational sacs observed under ultrasound at 4–5 weeks/total no. of embryos transferred) × 100%; Biochemical pregnancy rate = (no. of cycles with biochemical pregnancy/total no. of embryo transfer cycles) × 100%; Clinical pregnancy rate = (no. of clinical pregnancies/no. of transfer cycles) × 100%; Miscarriage rate = (no. of miscarriages before 22 weeks/no. of clinical pregnancies) × 100%.

However, regarding the live birth rate per transfer cycle, although the positive group exhibited a lower rate (21.3% vs. 29.9%, p=0.143), the difference did not reach statistical significance. Additionally, there were no significant differences in clinical pregnancy rates, embryo implantation rates or biochemical pregnancy rates per transfer cycle between the two groups.

### 3.4 Binary logistic regression for live birth rate and miscarriage rate per transfer, and multivariate Cox regression model for CLBR

**Figure 3:**
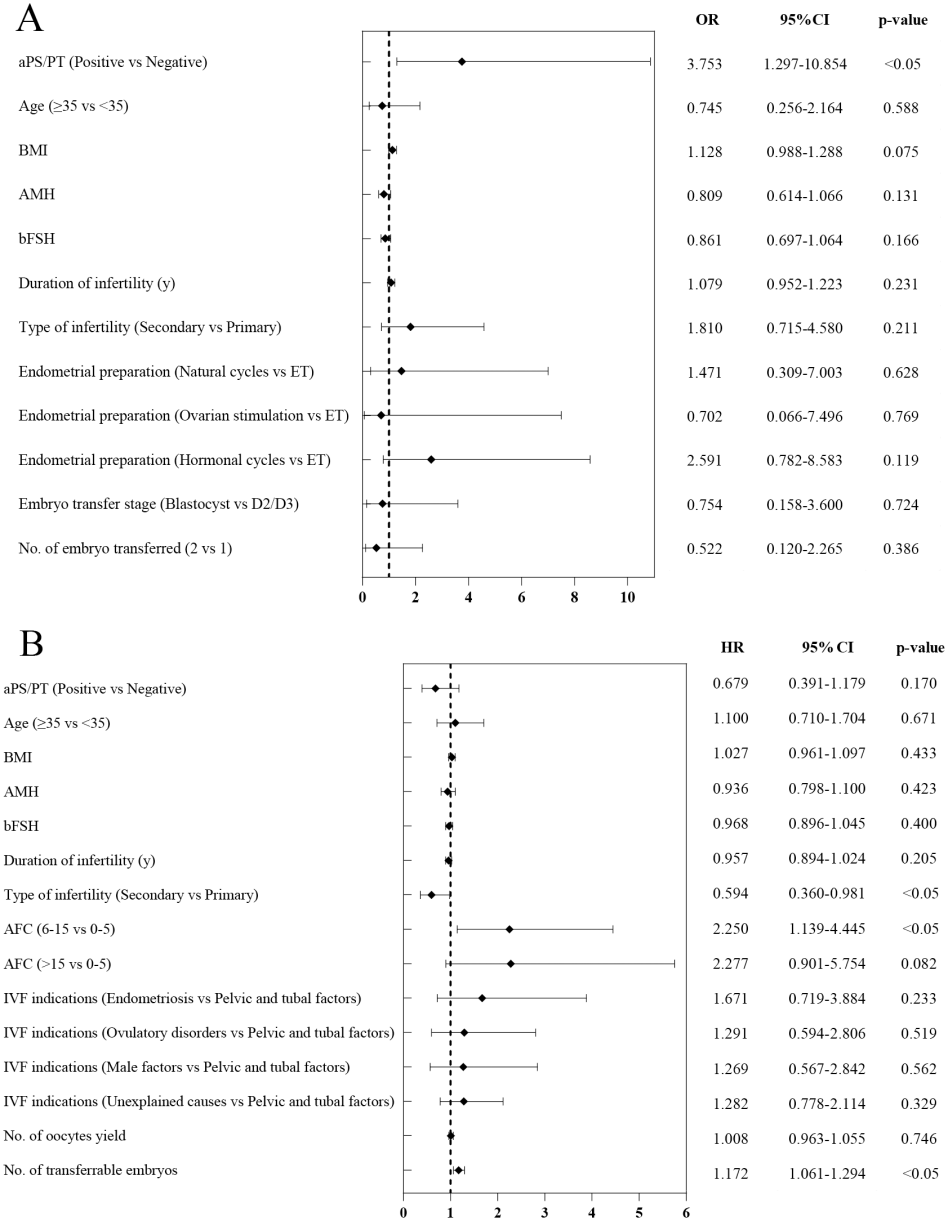
Binary logistic regression analysis of risk factors for miscarriage rate per transfer cycle **(A)** and cox regression model for predicting risk factors for **(B)** cumulative live birth per stimulation cycle. Abbreviations: aPS/PT, Anti-Phosphatidylserine/Prothrombin antibodies; AMH, Anti-Müllerian Hormone; BMI, Body Mass Index; AFC, Antral Follicle Count; OR, Odds Ratio; HR, hazard ratio; CI, confidence interval.

Binary logistic regression analysis was performed to assess the impact of aPS/PT positivity on the live birth rate and the miscarriage rate per transfer cycle. After adjusting for confounding factors, no statistically significant difference was observed in the live birth rate per transfer cycle between the aPS/PT-positive and negative groups (OR (95% CI) = 0.637 (0.331-1.226), p=0.177) (**Supplementary Figure S1**). However, for the miscarriage rate, aPS/PT positivity may be an independent risk factor. Notably, the probability of miscarriage in the positive group was 3.753 times higher than that in the negative group (OR (95% CI) = 3.753 (1.297-10.854), p<0.05) (**Figure 3A**). A Cox proportional hazards model was used to analyze the effect of aPS/PT on the cumulative live birth rate (adjusted for age, BMI, AMH, basal FSH, infertility duration, infertility type, antral follicle count, IVF indication, number of oocytes retrieved, and number of transferable embryos). After adjusting for relevant confounding factors, no significant association was found between aPS/PT and the cumulative live birth rate (HR (95% CI) = 0.685 (0.395-1.190), p=0.180) (**Figure 3B**).

## 4 Discussion

In this study, we investigated the impact of anti-phosphatidylserine/prothrombin antibody (aPS/PT) on IVF/ICSI outcomes in the unexplained recurrent implantation failure (URIF) population. Our findings indicate that aPS/PT positivity may serve as an independent risk factor for miscarriage in URIF patients (OR = 1.938, 95% CI: 1.002-3.749, p<0.05). Additionally, no statistically significant differences were detected between the aPS/PT-positive and negative groups in terms of oocyte quality, embryo parameters, clinical pregnancy rate, biochemical pregnancy rate, or live birth rate per transfer cycle, and risk modeling revealed no significant associations between aPS/PT status and the cumulative live birth rate.

The primary objective of a successful controlled ovarian stimulation cycle is to obtain 8–14 high quality oocytes, which can provide 2–3 embryo transfer opportunities, this is crucial for improving the success rate of embryo transplantation. Previous studies have indicated that autoantibodies may be associated with ovarian inflammatory responses and diminished ovarian reserve. Systemic lupus erythematosus (SLE) can trigger systemic inflammation, including autoimmune oophoritis, which may subsequently impair ovarian function (19). Furthermore, a retrospective study of 96 cohorts with diminished ovarian reserve demonstrated statistically heightened concentrations of anti-Jo-1 antibodies and proteinase 3 (20). A retrospective study by Yue Sun et al. revealed that thyroid peroxidase antibodies (TPOAb) > 100 IU/ml combined with thyroid-stimulating hormone (TSH) >2.5 mIU/L may impact the functional ovarian reserve of infertile individuals (21). As a type of autoantibody, aPS/PT-positive women may exhibit a proinflammatory state that potentially affects oocyte quality. Previous research by Liu et al. confirmed that aPS/PT-positive patients presented lower oocyte yields and fertilization rates (22). Unlike previous investigations, our study specifically focused on oocyte retrieval cycles in patients with unexplained recurrent implantation failure (URIF) and utilized propensity score matching (PSM) to control for confounding variables. The results revealed no significant differences in the oocyte or embryo parameters between the aPS/PT-positive and negative groups (**Supplementary Table S1**). These findings suggest that aPS/PT IgG/IgM antibodies may not impair ovarian responsiveness or embryo quality in URIF patients.

Previous studies have indicated that autoantibodies may adversely affect female reproductive health, particularly in the maintenance of early pregnancy. Antiphospholipid antibodies (aPLs) can induce placental micro thrombosis and decidual vascular necrosis, resulting in inadequate placental perfusion and ultimately leading to placental formation failure or embryo implantation defects (23). As a component of aPLs, aPS/PT are related to various clinical manifestations, such as thrombosis and gestational adverse events in patients with anti-phospholipid Syndrome (APS) (24). Furthermore, aPS/PT IgG and IgM can induce pregnancy complications through local placental thrombosis and the inflammatory response (25). However, overall, different studies have shown divergent views on the role of aPS/PT in reproductive outcomes. A Chinese cohort study demonstrated that aPS/PT IgM has superior diagnostic sensitivity for early recurrent pregnancy loss and unexplained miscarriage in patients with obstetric antiphospholipid syndrome (OAPS) compared with other aPL profiles (26). Additionally, a retrospective study involving 186 Chinese APS patients indicated that aPS/PT has high performance in APS diagnosis, especially when standard antibody tests yield negative results, and serves as a predictor for venous thrombosis and fetal loss (16). Conversely, the 2023 report from the International Society on Thrombosis and Hemostasis Scientific and Standardization Committee on Lupus Anticoagulant/Antiphospholipid Antibodies indicated that, compared with other aPLs, aPS/PT provides limited benefit to established OAPS diagnosis, but may have greater value in late-stage miscarriages in OAPS (25). However, previous studies have not excluded interference from other antibodies. For example, research on aPS/PT in OAPS pregnancy complications has only compared the risk levels of aPS/PT and standard antibodies, without directly illustrating the specific role of aPS/PT in pregnancy outcomes and complications. Furthermore, although considerable research has been conducted on aPS/PT, all the aforementioned studies have focused on its role in the post implantation embryo maintenance phase, with no dedicated exploration of its impact during the implantation window or on specific populations with URIF. Furthermore, well-established clinical treatment protocols currently exist for definitive obstetric antiphospholipid syndrome (OAPS), such as the combination of prophylactic low-molecular-weight heparin with low-dose aspirin (27). Additional studies have reported that the adjunctive use of hydroxychloroquine during pregnancy in patients with antiphospholipid syndrome significantly reduces the pregnancy loss rate (from 81% to 19%, p < 0.05) (28). However, for patients with non-criteria obstetric antiphospholipid syndrome (NC-OAPS, defined by the presence of non-criteria antiphospholipid antibodies alongside adverse pregnancy events), no approved specific treatment regimen is currently available. The prevailing clinical recommendation is to initiate low-dose aspirin therapy from early pregnancy to prevent preeclampsia (29). Notably, more than half of such patients in clinical practice are still managed according to the standard OAPS protocol, which involves preconceptional low-dose aspirin combined with prophylactic low-molecular-weight heparin (30). Therefore, conducting in-depth research on this patient population exhibiting only non-criteria antibody positivity is of significant importance. To control potential confounding factors and ensure cohort homogeneity, this study enrolled URIF patients who tested negative for standard antibodies and other autoimmune diseases. The results demonstrated that the aPS/PT-positive group had a significantly higher miscarriage rate per transfer cycle (41.4% vs 20.4%, p<0.05) and a significantly lower cumulative live birth rate per oocyte retrieval cycle (20.0% vs 32.1%, p<0.05) compared to the negative group. Although Kaplan-Meier survival curves revealed significant differences in cumulative live birth rates between the aPS/PT-positive and negative groups (HR (95% CI) = 0.604 (0.382-0.956), p<0.05), Cox proportional hazards models revealed no significant associations between aPS/PT and cumulative live birth rates. This finding suggests that the observed difference in the Kaplan-Meier survival curves may be attributed to confounding factors (such as age, BMI, AMH, etc.), resulting in biased estimates. Consequently, after adjusting for these confounders in the Cox proportional hazards model, anti-phosphatidylserine/prothrombin antibodies showed no significant association with the cumulative live birth rate per oocyte retrieval cycle. However, binary logistic regression models adjusted for confounding factors demonstrated a significant correlation between aPS/PT and the miscarriage rate (OR (95% CI) = 3.753 (1.297-10.854), p<0.05). Based on these findings, we hypothesize that aPS/PT positivity may be an independent risk factor for increased miscarriage rates in URIF patients, but may not serve as a predictor of cumulative live birth outcomes in this population.

However, our study also has several limitations. 1) As a single-center retrospective study, the cohort may lack representativeness of broader populations, potentially compromising the external validity and generalizability of the findings. 2) Due to the inevitable incompleteness of retrospective information collection, data on possible immunomodulatory medications such as prednisone, hydroxychloroquine, and aspirin could not be analyzed and excluded. 3) Due to the limited sample size, we did not perform subgroup analyses on the basis of antibody concentration levels within the aPS/PT-positive group. Additionally, we did not differentiate between aPS/PT IgG-positive and IgM-positive cases. 4) Due to the design of retrospective studies, it is difficult to establish a clear causal relationship, and only associations can be described. 5) Variations in aPS/PT detection methods across different centers may limit the generalizability of the results. 6) Low statistical power prevents the analysis of obstetric complications, such as gestational hypertension (GHTN) and gestational diabetes mellitus (GDM). Comprehensive multicenter studies are required to confirm the pathogenic function of aPS/PT.

## 5 Conclusion

In conclusion, this study focused on the impact of aPS/PT in URIF patients. The results demonstrated that aPS/PT may not adversely affect oocyte quality or embryo quality in URIF patients, but may serve as an independent risk factor for miscarriage (OR = 1.938, 95% CI: 1.002-3.749, p<0.05). This finding addresses a critical knowledge gap regarding the clinical relevance of aPS/PT in URIF. We recommend increased clinical attention for aPS/PT-positive URIF patients, and consultation with rheumatology and immunology specialists may be necessary to determine whether close monitoring and treatment are needed. Similarly, more follow-up data and therapeutic information may positively impact live birth outcomes in URIF patients. Further research (large-scale prospective clinical studies) is needed to address the aforementioned limitations.

## Data Availability

The raw data supporting the conclusions of this article will be made available by the authors, without undue reservation.

## Supplementary material

Supplementary Figure 1 Binary logistic regression analysis of risk factors for live birth per transfer cycle. Abbreviations: aPS/PT, Anti-Phosphatidylserine/Prothrombin antibodies; AMH, Anti-Müllerian Hormone; BMI, Body Mass Index; AFC, Antral Follicle Count; OR, Odds Ratio; CI, confidence interval.

Supplementary Table 1 Cycle stimulation characteristics after propensity score matching. Continuous variables were presented as median-interquartile range, categorical variables were expressed as number (percentage), p< 0.05 was considered statistically significant. Fertilization rate = (no. of fertilized oocytes/total no. of oocytes retrieved) × 100%; 2PN rate = (no. of 2PN embryos/no. of MII oocytes) × 100%; Cleavage rate = (no. of cleaved embryos/total no. of fertilized oocytes) × 100%; High-quality embryos rate = (no. of high-quality embryos/total no. of embryos) × 100%; 2PN: 2 Pronuclen.
