# Supplementary figures and images for: The impact of anti-phosphatidylserine/prothrombin antibodies on pregnancy outcomes in patients with unexplained recurrent implantation failure: a retrospective cohort study
Source: Front Immunol. 2026 Jan 21;17:1731905. doi: 10.3389/fimmu.2026.1731905 (PMC12868217; doi:10.3389/fimmu.2026.1731905)

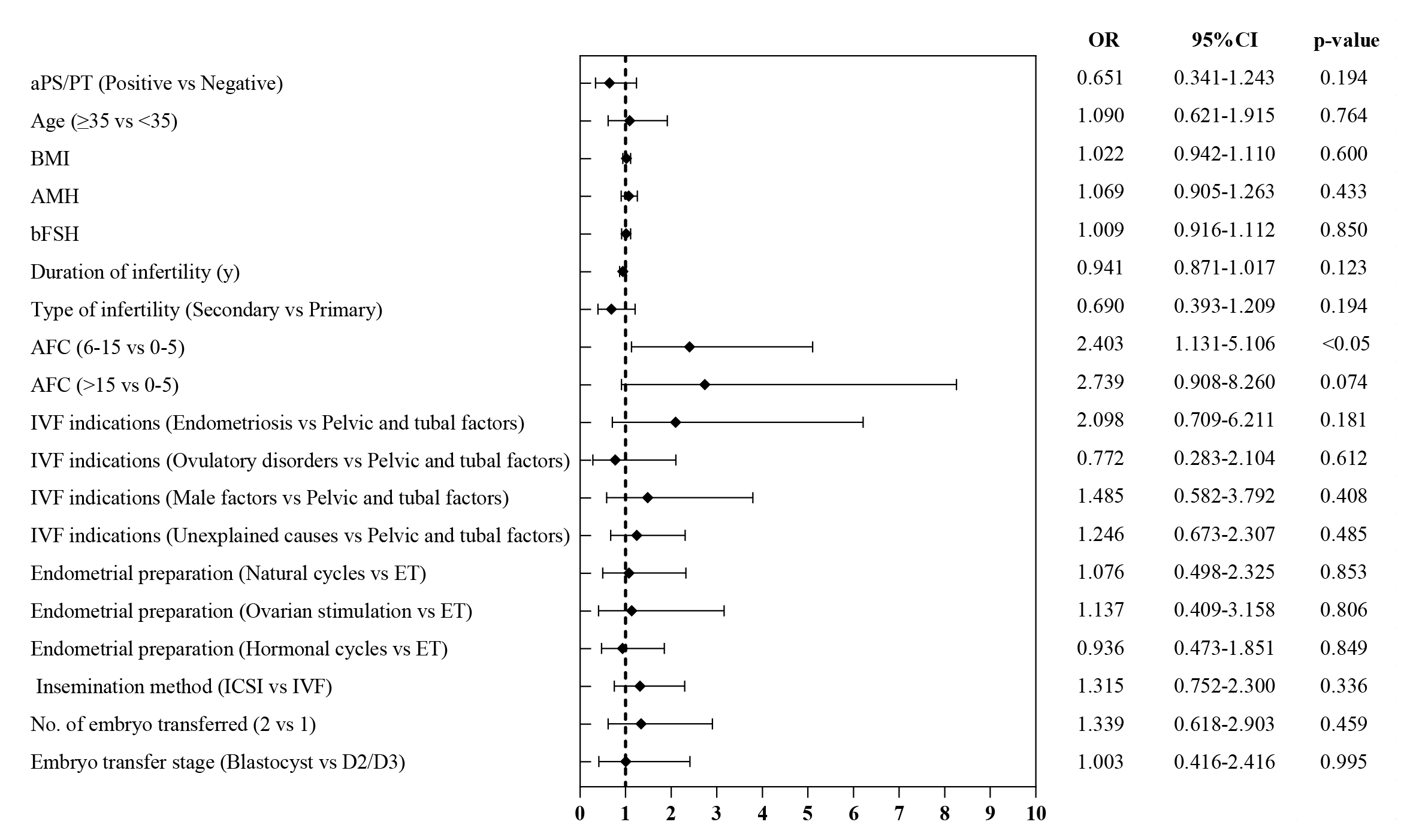

Supplement: Supplementary Figure 1 — Binary logistic regression analysis of risk factors for live birth per transfer cycle. Abbreviations: aPS/PT, Anti-Phosphatidylserine/Prothrombin antibodies; AMH, Anti-Müllerian Hormone; BMI, Body Mass Index; AFC, Antral Follicle Count; OR, Odds Ratio; CI, confidence interval. [file Image1.tif]
