# Supplementary material for: The impact of anti-phosphatidylserine/prothrombin antibodies on pregnancy outcomes in patients with unexplained recurrent implantation failure: a retrospective cohort study
Source: Front Immunol. 2026 Jan 21;17:1731905. doi: 10.3389/fimmu.2026.1731905 (PMC12868217; doi:10.3389/fimmu.2026.1731905)
Supplement: Supplementary Table 1 — Cycle stimulation characteristics after propensity score matching. Continuous variables were presented as median-interquartile range, categorical variables were expressed as number (percentage), p< 0.05 was considered statistically significant. Fertilization rate = (no. of fertilized oocytes/total no. of oocytes retrieved) × 100%; 2PN rate = (no. of 2PN embryos/no. of MII oocytes) × 100%; Cleavage rate = (no. of cleaved embryos/total no. of fertilized oocytes) × 100%; High-quality embryos rate = (no. of high-quality embryos/total no. of embryos) × 100%; 2PN: 2 Pronuclen. [file Table1.docx]

**Supplemental Table 1: Cycle stimulation characteristics after propensity score matching.**

| **Characteristic** | **Antibody Positive Group (n=80)** | **Antibody Negative Group After PSM Matching (n=240)** | **P-Value** |  |
| --- | --- | --- | --- | --- |
| Ovulation induction protocol |  |  |  |  |
| Antagonist protocol (n%) | | 43 (53.8) | 128 (52.5) |  |
| Long protocol (n%) | 14 (17.5) | 48 (20.0) |  |  |
| Ultralong protocol (n%) | 13 (16.3) | 34 (14.2) | 0.877 |  |
| Short protocol (n%) | 7 (8.8) | 25 (10.4) |  |  |
| Other protocols (n%) | 3 (3.8) | 5 (2.9) |  |  |
| Days of Gn (days) | 11 (9-12) | 11 (9-12) | 0.894 |  |
| Total Gn dosage (IU) | 3188 (2250-3970) | 3038 (2250-3900) | 0.709 |  |
| Cancellation rate, n (%) | 12 (15.0) | 33 (13.8) | 0.781 |  |
| No. of oocytes yield | 8.5 (4-13) | 9 (5-13) | 0.615 |  |
| No. of oocytes fertilized | 5.5 (3.0-9.0) | 6.0 (3.0-9.8) | 0.718 |  |
| Fertilization rate, % (n) | 76.5 (515/673) | 76.0 (1593/2096) | 0.783 |  |
| 2PN | 4.5 (2.3-7.8) | 5.0 (2.0-8.0) | 0.667 |  |
| 2PN rate, % (n) | 58.1 (429/738) | 54.7 (1266/2313) | 0.106 |  |
| No. of cleaved embryos | 5.0 (3.0-8.8) | 6.0 (3.0-9.8) | 0.673 |  |
| Cleavage rate, % (n) | 96.9 (499/515) | 98.2 (1565/1593) | 0.063 |  |
| No. of high-quality embryos | 3.0 (1.3-5.0) | 3.0 (1.0-5.0) | 0.997 |  |
| High-Quality Embryos rate, % (n) | 58.1 (290/499) | 56.6 (886/1565) | 0.555 |  |
| No. of transferrable embryos | 2 (1-3) | 2 (1-3) | 0.636 |  |

Continuous variables were presented as median-interquartile range, categorical variables were expressed as number (percentage), p< 0.05 was considered statistically significant.

Fertilization rate = (no. of fertilized oocytes / total no. of oocytes retrieved) × 100%; 2PN rate = (no. of 2PN embryos / no. of MII oocytes) × 100%; Cleavage rate = (no. of cleaved embryos / total no. of fertilized oocytes) × 100%; High-quality embryos rate = (no. of high-quality embryos / total no. of embryos) × 100%; 2PN: 2 Pronuclen.
