# Supplementary material for: The impact of anti-phosphatidylserine/prothrombin antibodies on pregnancy outcomes in patients with unexplained recurrent implantation failure: a retrospective cohort study
Source: Front Immunol. 2026 Jan 21;17:1731905. doi: 10.3389/fimmu.2026.1731905 (PMC12868217; doi:10.3389/fimmu.2026.1731905)
Supplement: Supplementary file 3 [file DataSheet1.docx]

**Supplemental Figure 1: Binary logistic regression analysis of risk factors for live birth per transfer cycle.**

Abbreviations: aPS/PT: Anti-Phosphatidylserine/Prothrombin antibodies; AMH: Anti-Müllerian Hormone; BMI: Body Mass Index; AFC: Antral Follicle Count; OR: Odds Ratio; CI: confidence interval.
